# Serum soluble triggering receptor levels expressed on myeloid cells2 identify early acute kidney injury in infants and young children after pediatric cardiopulmonary bypass

**DOI:** 10.3389/fped.2023.1185151

**Published:** 2023-06-26

**Authors:** Mingwei Sun, Lijun Yang, Qing Zong, Liyang Ying, Xiwang Liu, Ru Lin

**Affiliations:** ^1^ Department of CPB/ECMO, National Clinical Research Center for Child Health, The Children's Hospital, Zhejiang University School of Medicine, Hangzhou, China; ^2^ Department of Heart Center, National Clinical Research Center for Child Health, The Children's Hospital of Zhejiang University School of Medicine, Hangzhou, China

**Keywords:** acute kidney injury, cardiopulmonary bypass, soluble TREM2, prognostic factors, young children

## Abstract

**Background:**

Acute kidney injury (AKI) is a potential complication after cardiopulmonary bypass (CPB) of pediatric cardiac surgery and contributes to a certain amount of perioperative mortality. Serum soluble triggering receptor expressed on myeloid cells2 (sTREM2) is an inflammation-associated cytokine in circulation. Alterations of sTREM2 level have been reported in Alzheimer's disease, sepsis, and some other pathologic conditions. This study aimed to investigate the role of sTREM2 as a forecasting factor for AKI in infants and young children and other factors associated with early renal injury after pediatric CPB.

**Methods:**

A prospective cohort study with consecutive infants and young children ≤ 3 years old undergoing CPB from September 2021 to August 2022 was conducted in an affiliated university children's hospital. These patients were divided into an AKI group (*n* = 10) and a non-AKI group (*n* = 60). Children′s characteristics and clinical data were measured. Perioperative sTREM2 levels were analyzed with enzyme-linked immunosorbent assay (ELISA).

**Results:**

In children developing AKI, the sTREM2 levels significantly decreased at the beginning of CPB compared to the non-AKI group. Based on binary logistic regression analysis and multivariable regression analysis, risk-adjusted classification for congenital heart surgery (RACHS-1), operation time, and the s-TREM2 level at the beginning of CPB (AUC = 0.839, *p* = 0.001, optimal cut-off value: 716.0 pg/ml) had predictive value for post-CPB AKI. When combining the sTREM2 level at the beginning of CPB and other indicators together, the area under the ROC curve enlarged.

**Conclusions:**

Operation time, RACHS-1 score, and sTREM2 level at the beginning of CPB were independent prognosis factors of post-CPB AKI in infants and young children ≤ 3 years old. Decreased sTREM2 identified post-CPB AKI, and ultimately hampered the outcomes. Our findings indicated that sTREM2 may be a protective factor for AKI after CPB in infants and young children ≤ 3 years old.

## Introduction

AKI after CPB in infants and young children is one of the known risk factors that lead to hospital morbidity and mortality (1). Previous reports have identified some predictive risk factors (2), but when evaluating perioperative AKI, serum creatinine (sCr) remains the most commonly used indicator. Its performance does not change until half of the kidney function is damaged (3). In order to detect early-stage AKI and prediction of adverse clinical outcomes, new biomarkers are being studied in recent years, among them, both cystatin c and kidney injury molecule-1 (KIM-1) have shown some performance after CPB (4).

Studies have shown that the triggering receptor expressed on myeloid cell-2 (TREM2) is a kind of receptor with inflammatory regulation function on the surface of various immune cell membranes, and it is expressed in multiple organs. In a previous study of Alzheimer's disease, TREM2 was shown to help maintain the normal metabolism of microglia (5) and it also took part in metabolic coordination between macrophages and hepatocytes (6). It is currently thought to be an anti-inflammatory factor. Soluble TREM2 (sTREM2) of myeloid cells is the expression form of TREM2 in plasma. In a recent Alzheimer's disease study, it was shown to induce neuron protection by activating the innate immune system (7).

In our study, we conducted a prospective clinical study in infants and young children ≤ 3 years old with CPB during the perioperative period. Our aim was to explore the association between sTREM2 levels and the risk of post-CPB AKI and then elucidate the prognostic value of s-TREM2 levels for post-CPB AKI.

## Materials and methods

### Design and population

A cohort study with consecutive infants and young children ≤ 3 years old undergoing CPB from September 2021 to August 2022 was conducted in an affiliated university children's hospital. This was a single-center and prospective study. Exclusion criteria included: being >3 years old, pre-existing renal insufficiency or heart failure (potential renal hypoperfusion), major chromosomal abnormalities, tumor or other chronic diseases, palliative cardiac surgery, and application of nephrotoxic drugs. AKI was diagnosed by serum creatinine (sCr) according to the KDIGO (Kidney Disease: Improving Global Outcomes) criteria. An AKI group and a non-AKI group were compared based on post-CPB AKI. Data were derived from the medical record system of the cardiac intensive care unit (CICU).

For each patient, we collected their weight, age, risk-adjusted classification for congenital heart surgery (RACHS-1), perioperative levels of serum creatinine and cystatin c, operation time (including CPB time and aortic cross-clamp time), mechanical ventilation time, days of ICU and hospital stay. Before, in the beginning, at the end of CPB, and one hour and six hours after post-operation, 2 ml of blood samples were collected. After being centrifuged at 2,500 rpm at 4 °C for 5 min, we took the plasma samples and then stored them at−80 °C. S-TREM2 levels were measured by an enzyme-linked immunosorbent assay (ELISA) kit (ELK, Wuhan, China). Serum creatinine samples were collected 24 h after CPB, serum cystatin c samples were collected 1 h after CPB, and they were both measured by an auto-biochemistry analyzer (Modular Analytics, Roche, USA).

### CPB management

Endotracheal intubation, intravenous aspiration, and anesthesia combined with mild hypothermia (about 32–35°C) CPB were conducted during each reconstructive operation. During CPB, we maintained hematocrit levels at the beginning of CPB at approximately 25%–28%, and then conventional ultrafiltration plus modified ultrafiltration were used to maintain hematocrit levels at the end of CPB at approximately 35%–40%. When patients′ estimated nadir hematocrit was ≤ 25%, we primed the CPB circuit with packed red blood cells to achieve the goals mentioned above. Most patients were followed up in cardiac su during the perioperative period, almost 3 days after surgery, and then treated in the department of cardiac surgery until they were discharged from the hospital.

### Statistical analysis

SPSS V.25 (SPSS Inc, Chicago, IBM, USA) was used for statistical analysis. Categorical variables were analyzed using the *χ*2 test and were described as percentages. Continuous variables were analyzed using the Mann-Whitney *U* test and were described as median (IQR). Variables with *P* < 0.05 in the *χ*2 test or *U* test entered binary logistic regression and multivariate regression analysis models to identify the independent variables that individually might associate with AKI (*p* ≤ 0.05).

## Results

### Definition of AKI

In all, 83 patients who underwent elective pediatric cardiac surgery under CPB were included in this study. Of these, 13 patients were respectively excluded from our study because of prematurity (6), preoperative renal injury (3), major chromosomal abnormalities (2), and palliative cardiac surgery (2). SCr levels at 24 h after CPB were collected. AKI was defined as a ≥ 50% increase of sCr levels from baseline within 48 h after surgery according to KDIGO criteria, and the severity of AKI was classified into stages 1, 2, or 3 based on the maximal change in sCr from preoperative baseline levels. Of all the 70 patients enrolled, 10 (14.3%) developed post-CPB AKI according to KDIGO criteria, and these patients were respectively classified into stages 1 (7, 70%), 2 (2, 20%), and 3 (1, 10%). Only one patient received peritoneal dialysis, and none of the children required continuous hemodialysis.

### Demographics and clinical data

Children′s demographics and perioperative clinical data are shown in Table 1. Based on the primary outcomes, children in the AKI group were younger [53 (13–163) days vs. 204 (87–335) days, *P = *0.009] and lighter [3.95 (3.55–7.02) kg vs. 7.00 (4.85–8.92) kg, *P = *0.014], and they had higher RACHS-1 scores [3 vs. 2, *P *<* *0.001]. During the treatment, the AKI group underwent significantly longer cross-clamp time [86 (66.00–96.75) min vs. 46 (34.00–62.75) min, *P = *0.001], CPB time [120.00 (91.75–143.00) min vs. 68.00 (53.00–93.00) min, *P = *0.003], and operation time [173.00 (143.50–221.00) min vs. 130.00 (113.00–164.25) min, *P = *0.007]. When comparing the impact on clinical outcomes, the AKI group had a longer duration of mechanical ventilation [37.50 (24.75–82.00) h vs. 23.00 (7.25–35.00) h, *P *<* *0.001], a longer length of stay in the ICU [7.00 (5.00–10.00) day vs. 5.00 (4.00–5.75) day, *P = *0.005], and a longer hospital stay [26.00 (16.00–35.25) day vs. 15.50 (12.25–20.75) day, *P = *0.003] than the non-AKI group. No difference was found in hematocrit level, ultra-filtrated volume, and intraoperative fluid volume during CPB between the two groups. In the cohort of children in this study, serum cystatin c levels were significantly increased both before [1.41 (1.19–1.86) mg/L vs. 1.14 (0.91–1.28) mg/L, (*P = *0.014)] and after CPB in AKI children [1.18 (1.13–1.46) mg/L vs. 0.85 (0.77–1.04) mg/L, (*P = *0.001)].

**Table 1:** Patient demographics and clinical data.

Variable (pre/intraoperative)	AKI		Non-AKI		
	(*n *= 10) Median	(IQR)/%	(*n *= 60) Median	(IQR)/%	*P*
Age (days)	53	13−163	204	87–335	0.009*
Weight (kg)	3.95	3.55–7.02	7.00	4.85–8.92	0.014*
CPB time (min)	120.00	91.75–143.00	68.00	53.00–93.00	0.003*
Cross-clamp time (min)	86.00	66.00–96.75	46.00	34.00–62.75	0.001*
Operation time (min)	173.00	143.50–221.00	130.00	113.00–164.25	0.007*
RACHS-1 score	3	2–4	2	2	<0.001*
Ultrafiltrated volume (ml)	500	300–662.5	400.0	300.0–492.5	0.120
Intraoperative fluid volume (ml)	85	−60–177.5	20	−30–80	0.334
Hematocrit during CPB(%)	27.0	25.75–28.1	26.8	25.20–28.00	0.750
Preoperative cystatin c (mg/L)	1.41	1.19–1.86	1.14	0.91–1.28	0.014*
Post-CPB cystatin c (mg/L)	1.18	1.13–1.46	0.85	0.77–1.04	0.001*
Mechanical ventilation time(h)	37.50	24.75–82.00	23.00	7.25–35.00	<0.001*
ICU stay (days)	7.00	5.00–10.00	5.00	4.00–5.75	0.005*
Hospital stay (days)	26.00	16.00–35.25	15.50	12.25–20.75	0.003*

AKI, acute kidney injury; CPB cardiopulmonary bypass; RACHS-1, risk-adjusted classification for congenital heart surgery; ICU, intensive care unit.

Data were presented as median (IQR).

*P < 0.01.

### Perioperative levels of s-TREM2

**Figure 1:**
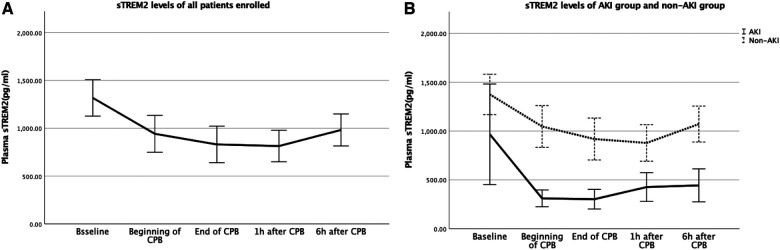
(**A**) Baseline levels and changes of sTREM2 for all the patients; (**B**) baseline levels and changes of sTREM2 for the patients in the AKI group and non-AKI group. AKI, acute kidney injury; sTREM2, serum soluble triggering receptor expressed on myeloid cells2.

We evaluated perioperative sTREM2 levels at the time t1-t5 (baseline, beginning of CPB, end of CPB, 1 h after CPB, and 6 h after CPB) to find the effects of CPB on sTREM2 levels after cardiac surgery (Table 2). In all children enrolled, sTREM2 levels began to decrease at the beginning of CPB and reached the nadir level 1 h after CPB. During the 24 h after CPB, it gradually returned but was still below baseline 6 h after the operation. Before the operation, sTREM2 levels were similar between the AKI and non-AKI groups. As compared with the non-AKI group, sTREM2 levels decreased significantly when CPB started. In addition, the sTREM2 levels did not significantly increase within 6 h after the operation in the AKI group (Figures 1A,B).

**Table 2:** Comparison of perioperative sTREM2 levels between the two groups.

sTREM2 levels (pg/ml)	Total	AKI (*n *= 10)	Non-AKI (*n *= 60)	*P*
Baseline (t1)	1,317.30 ± 179.84	967.04 ± 520.52	1,375.67 ± 301.25	0.202
Beginning of CPB (t2)	941.69 ± 181.14	310.58 ± 79.86	1,046.87 ± 330.12	0.001*
End of CPB (t3)	830.53 ± 179.98	302.27 ± 104.45	918.57 ± 330.61	0.003*
1 h after CPB (t4)	813.94 ± 169.09	426.56 ± 205.69	878.50 ± 272.58	0.014*
6 h after CPB (t5)	977.30 ± 170.73	443.70 ± 236.15	1,071.83 ± 314.18	0.007*

sTREM2, serum soluble triggering receptor expressed on myeloid cells2; AKI, acute kidney injury; CPB cardiopulmonary bypass.

Data were presented as mean ± SD.

*P < 0.01.

### Predictors of post-CPB AKI

**Figure 2:**
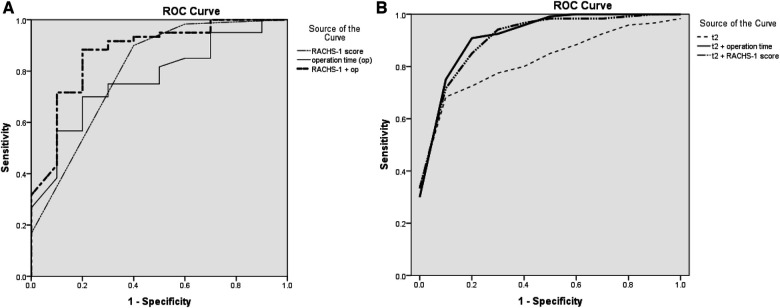
(**A**) ROC curve for RACH-1 score, operation time, and the combination of RACH-1 score and operation time to predict post-CPB AKI; (**B**) ROC curve for sTREM2 level at t2, the combination of sTREM2 level at t2 with RACH-1 score, and the combination of sTREM2 level at t2 with operation time to predict post-CPB AKI. ROC, receiver operating characteristic; t2, time 2 (beginning of CPB); RACHS-1, risk-adjusted classification for congenital heart surgery.

Analysis of AKI and non-AKI variables was generalized in Table 3 to predict post-CPB AKI. In univariate logistic analysis, operation time (*p* = 0.032), RACHS-1 score (*p* = 0.024), and sTREM2 level of t2 (*p* = 0.028) were associated with post-CPB AKI. In multivariate regression, operation time (*p* = 0.019), RACHS-1 score (*p* = 0.002), and sTREM2 level of t2 (*p* = 0.038) remained as independent predictors (Table 3). Thus, the sTREM2 level at the beginning of CPB was an independent prognosis factor for post-CPB AKI. To substantiate the prognosis accuracy and then find an optimal cut-off value, ROC curves were constructed. The sensitivity and specificity of the RACHS-1 score (AUC = 0.798, *p* = 0.003), operation time (AUC = 0.768, *p* = 0.007), and their combination (AUC = 0.872, *p* <* *0.001) are shown in Figure 2A. Then, we introduced the sTREM2 level (at the beginning of CPB, t2) into this model, and the prognosis accuracy improved. Sensitivity and specificity of the sTREM2 level at the beginning of CPB (t2 in Figure 2B. AUC = 0.839, *p* = 0.001, optimal cut-off value: 716.0 pg/ml), t2+ RACHS-1 score (Figure 2B. AUC = 0.925, *p *<* *0.001), and t2 + operation time (Figure 2B. AUC = 0.933, *p *<* *0.001) are shown in Figure 2B.

**Table 3:** Binary logistic models (forward stepwise) for causes of post-CPB AKI; multivariate regression models for predictors of post-CPB AKI.

	Univariate logistic analysis			Multivariate regression analysis		
	OR	95% CI	*P*	B	t	*P*
Age (day)			0.902			
Weight (kg)			0.597			
CPB time (min)			0.599			
Cross-clamp time (min)			0.521			
Operation time (min)	0.962	0.928–0.997	0.032*	0.232	2.118	0.019*
RACHS-1 score	0.212	0.055–0.814	0.024*	0.362	3.213	0.002*
sTREM2 levels (pg/ml)						
Baseline (t1)			0.732			
Beginning of CPB (t2)	1.009	1.001–1.016	0.028*	−0.249	−2.396	0.038*
End of CPB (t3)			0.913			
1 h after CPB (t4)			0.997			
6 h after CPB (t5)			0.565			

CPB, cardiopulmonary bypass; RACHS-1, risk-adjusted classification for congenital heart surgery; sTREM2, serum soluble triggering receptor expressed on myeloid cells2; OR, odds ratio; CI, confidence interval.

*P < 0.01.

## Discussion

### Main findings

Our study prospectively analyzed the risk factors of AKI after cardiac surgery under CPB in infants and young children. We found that weight, age, RACHS-1 score, CPB time, aortic cross-clamp time, and operation time had significant differences between the two groups, and then post-CPB AKI hampered the clinical outcome. Univariate logistic analysis and multivariate regression analysis showed that the RACHS-1 score, operation time, and the sTREM2 level at the beginning of CPB were independent prognosis factors for post-CPB AKI. When combining these indicators, we obtained better predictive efficiency. For example, when the RACHS-1 score and the sTREM2 level at the beginning of CPB (t2) jointly predicted post-CPB AKI, the discrimination ability improved to an AUC of 0.925 (*p* <* *0.001).

### Prognosis value of sTREM2

At present, peak SCr level is the most widely used independent predictor of post-CPB AKI, and it usually increases 2–3 days after AKI (3). According to the KDIGO-AKI rating, it is important to pay close attention to renal functional changes during pediatric mechanical circulatory support (MCS), even if the changes are very slight (8). Early identification of the signs and severity of post-CPB AKI is essential for improving prognosis. When there is a need to identify AKI earlier, sCr is not a timely indicator. To address this problem, biomarkers including cystatin c, neutrophil gelatinase-associated lipocalin (NGAL), kidney injury molecule-1 (KIM-1), and interleukin (IL)-18 have been discovered (9), but none of them showed signs as early as CPB started. To match or compare the prognosis value of sTREM2 with current predictors of early kidney injury, cystatin c samples were also collected before and after CPB. However, for some reason, the baseline levels of serum cystatin c in the AKI and non-AKI groups were significantly different in our study, thus, this star biomarker did not show sufficient clinical diagnosis value. As far as we know, our study is the first one focusing on the prognosis value of sTREM2 for post-CPB AKI in infants and young children. In our study, the s-TREM2 levels in the AKI group were significantly lower compared to the non-AKI group as early as the beginning of CPB and remained at low levels until 6 h after CPB. As an independent prognosis factor for post-CPB AKI, the sTREM2 level at the beginning of CPB (t2) when combined with other factors enhanced their prognosis accuracy, and all these observations allowed us to advance the awareness of post-CPB AKI to the beginning of CPB.

### Protective role of TREM2/sTREM2 in inflammation responses

Studies have found TREM2 predominantly expressed in macrophages, monocyte-derived dendritic cells, osteoclasts, and microglia (10). TREM2 is also reported as a soluble form (sTREM2) into the extracellular space when releasing its ectodomain and plays a regulating role (11). STREM2 was reported to trigger microglial activation inducing inflammatory responses and promoting survival (12). Cleavage of TREM2 leads to the release of sTREM2 through the proteolytic ectodomain shedding process (13). In this process, there may be several mechanisms involved in the regulation of sTREM2 shedding and sTREM2/TREM2-mediated cellular functions. The influence of sTREM2 itself and shedding effects on downstream TREM2 receptor signaling are not fully understood, but recent research might speculate that sTREM2 shedding occurs to modulate receptor activity (14), thus, levels of sTREM2 may reflect the degree of engagement of TREM2 receptor with its ligand (15).

### STREM2 may protect against post-CPB AKI

In previous studies, hypo-perfusion and inflammation during CPB might further result in post-CPB AKI. Hypo-perfusion and ischemia lead to vascular endothelium injury and activation in multiple organs. The contact between the human body and the non-physiological circuit during CPB triggers an aseptic inflammatory response. Over the last few years, TREM/sTREM2 have cleared their essential roles in the regulation of inflammatory responses in different tissues. One of the most well-known roles is their protective effect on the nervous system. TREM2/sTREM2 sustain cellular energetic and biosynthetic metabolism and then promote microglial responses during Alzheimer's disease, which maintains microglial metabolic fitness in Alzheimer's disease (16). Through the aspect of post-ischemic inflammatory response and neuronal apoptosis, TREM2 protects against cerebral ischemia/reperfusion injury in ischemic stroke (17). In other tissues and organs, TREM2 was found to promote the transition from the pro-inflammatory phase to the tissue repair phase by driving the acquisition of restorative properties in phagocytic macrophages, which revealed the controlling of phenotypic shifts in liver macrophages and impacts endothelial cell differentiation during tissue recovery (18). A recent study found that TREM2 finely modulates the IL-23/IL-17A immune pathway to prolong survival and improve organ injury from sepsis in aged mice (19). Based on these observations and the sTREM2/TREM2 regulating mechanisms mentioned above, we hypothesize that sTREM2 may modulate inflammatory responses and may play a protective role in post-CPB AKI. However, further studies are required to explore the underlining mechanisms.

Our study still has some limitations. First, this is a single-center prospective study, thus, the findings may not be universal in different heart centers; the findings may need to be validated in a multicenter clinical study. Second, the study was designed for young children (<3 years old), and most of their hematocrit at the end of CPB was maintained at a high level; we would like to investigate if these findings apply to older children. In addition, due to the sampling time points that mainly surrounded CPB, more data could not be collected after the children were transferred to CICU. In addition, the number of samples might have been insufficient, and thus we failed to directly compare the efficacy of s-trem2 with other early predictors of pediatric post-CPB AKI. Therefore, additional time points will be set in subsequent studies. Finally, the mechanism of this protective effect against AKI was unknown, and cell or animal studies are needed to find the corresponding signaling way and the relationship between sTREM2/TREM2 in the future.

## What is already known about this subject?

A soluble form of triggering receptor expressed on myeloid cells2 (sTREM2) exists in plasma and is generated from alternative splicing or receptor shedding. Binding an unknown receptor on other cells, sTREM2 may work together to regulate inflammation response with downstream TREM2. Preclinical and clinical studies have found that monitoring sTREM2 levels might guide the therapeutic implications in a variety of systems and diseases.

## What are the new findings?

Operation time, RACHS-1 score, and sTREM2 level at the beginning of CPB were independent prognosis factors of post-CPB AKI in infants and young children ≤ 3 years old. STREM2 in pediatric CPB patients may be a protective factor.

## What can we do in clinical practice in the future?

STREM2 may modulate inflammatory responses and may play a protective role in post-CPB AKI in infants and young children ≤ 3 years old. In the future, it may be a target of prevention and treatment in infants and young children for pediatric post-CPB AKI.

## Data Availability

The raw data supporting the conclusions of this article will be made available by the authors, without undue reservation.
